# Microwave-Assisted Functionalization of Multi-Walled Carbon Nanotubes for Biosensor and Drug Delivery Applications

**DOI:** 10.3390/pharmaceutics15020335

**Published:** 2023-01-19

**Authors:** Chanchal Kiran Thakur, Chandrabose Karthikeyan, Mariam Sami Abou-Dahech, Moawia Mohd A. M. Altabakha, Moayad Jamal Saeed Al Shahwan, Charles R. Ashby, Amit K. Tiwari, R. Jayachandra Babu, Narayana Subbiah Hari Narayana Moorthy

**Affiliations:** 1 Cancept Therapeutics Laboratory, Department of Pharmacy, Indira Gandhi National Tribal University, Lalpur, Amarkantak 484887, Madhya Pradesh, India; 2 Department of Pharmacology and Experimental Therapeutics, College of Pharmacy and Pharmaceutical Sciences, University of Toledo, Toledo, OH 43614, USA; 3 Department of Pharmaceutical Sciences, College of Pharmacy, Ajman University, Ajman P.O. Box 346, United Arab Emirates; 4 Center of Medical and Bio-Allied Health Sciences Research, Ajman University, Ajman P.O. Box 346, United Arab Emirates; 5 Department of Pharmaceutical Sciences, College of Pharmacy, St. John’s University, New York, NY 11431, USA; 6 Department of Cancer Biology, College of Medicine and Life Sciences, University of Toledo, Toledo, OH 43614, USA; 7 Department of Drug Discovery & Development, Harrison School of Pharmacy, Auburn University, Auburn, AL 36849, USA

**Keywords:** MWCNTs, microwave-assisted synthesis, microwave irradiation, graphene, vitamins, sustainable materials, drug delivery

## Abstract

Microwave-assisted synthetic methods have emerged as a popular technique for surface modification and the functionalization of multi-walled carbon nanotubes (MWCNTs) for diverse drug delivery applications. Microwave-induced functionalization of MWCNTs provides a high functionalization and requires less time than conventional techniques. Microwave methods are simple, fast, and effective for the covalent and noncovalent conjugation of MWCNTs with various biomolecules and polymers. The present review focuses on the synthetic and drug delivery applications of microwave irradiation techniques (MITs) for the functionalization of MWCNTs, using amino acids and other molecular frameworks containing amino groups, vitamins, proteins, epoxy moieties, metal nanoparticles, and polymers.

## 1. Introduction

**Figure 1:**
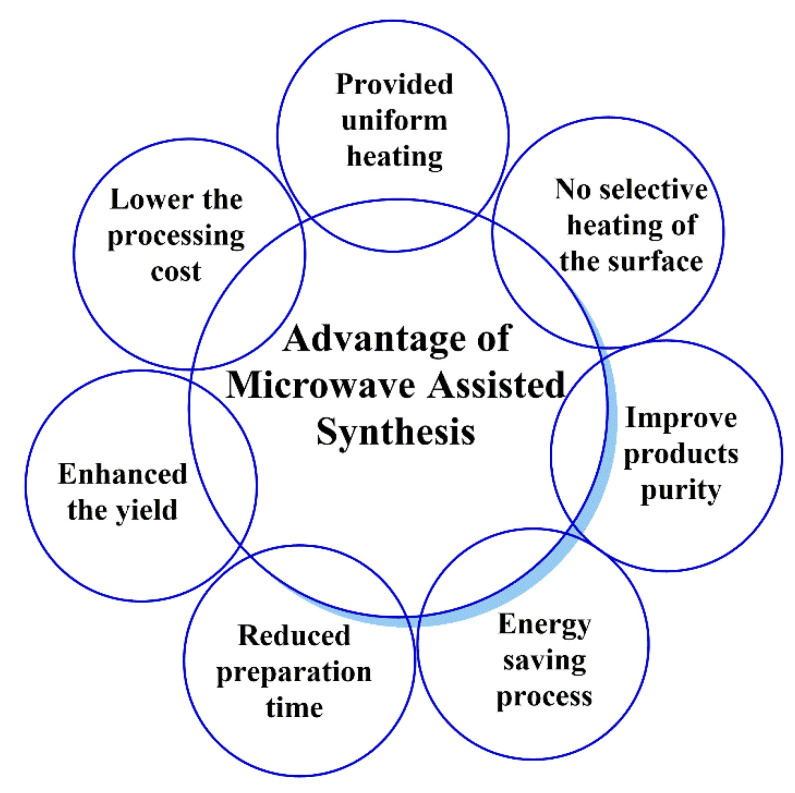
Basic advantages of microwave-assisted synthesis.

Microwave-assisted organic synthesis is a popular method in pharmaceutical and medicinal chemistry research due to its improved or increased productivity and sustainability [1,2]. Microwave-assisted synthesis is a simple, fast, and effective technology for linking ligands, polymers, peptides, and antibodies to nanocarriers with increased productivity and a shorter duration than conventional techniques [3]. Microwave chemistry has been recently used for the preparation of many nanocarriers for the targeted delivery of pharmaceuticals and the modification or functionalization of nanosystems for drug delivery [2,3,4]. Microwave chemistry is also used to increase the interactions/reactions between linker-nanotubes, ligand-nanoparticles, ligand-polymers, and cross-linked functionalization of drug molecules. We have summarized the additional advantages of microwave chemistry in Figure 1 [5].

### 1.1. Principle of Microwave Synthesis

In microwave heating, the charged molecules directly interact with the frequency of the electromagnetic radiation with wavelength ranging between 0.01 to 1 m. This creates reaction heat by either collision or a conductive process [6]. Microwave energy can change its polarity (from positive to negative) to produce heat by rapid orientation or reorientation of the reacting molecules involving dipolar and interfacial polarization mechanisms [5]. In the dipolar polarization mechanism, the polar molecules are more likely to be in a suitable frequency range (0.3–30 GHz) of heating in an electromagnetic field, which increases the polar interactions between polar molecules [6,7,8]. If the frequency range is too high, the interactions of polar molecules will be insufficient. In contrast, for polar molecules in the low-frequency range, more time will be required for interactions with other molecules [9]. Dipolar polarizations develop heat either by interacting with polar solvent molecules (e.g., ethanol, methanol, water) or polar solutes (e.g., ammonia, formic acid) [10]. Interfacial polarization, also known as space charge polarization, occurs when two molecules interact with each other, and this condition occurs when one material is dielectric and non-homogenous [9,11]. 

### 1.2. Microwave Absorption System

The microwave absorption system consists of three common electromagnetic waves (EMw): absorbed power, reflected power, and transmitted power [12]. The reflection power of EMw consists of multiple and surface reflections. Multiple reflections increase when the transmitted route of EMw is extended, increasing the absorbing capacity of EMw. Subsequently, microwaves provide heat to the compound at the interface of the electromagnetic field as EMw is converted into thermal energy. There are two basic methods that increase EMw absorption: (1) increasing the transmitted route of EMw in the absorbent by regulating the multilayer nanostructure and (2) modification of the electromagnetic parameters of EMw absorption [9,11].

Relative complex permittivity (*ε_r_* = *ε*’ − *jε*”) and relative complex permeability (*µ_r_* = *µ*’ − *jµ*”), are the two parameters that are essential for determining the microwave absorption mechanism, as *ε*’ and *µ*’ represent the association of energy storage, and *jε*” and *jµ*” represent the energy dissipation of a dipolar mechanism, conduction, resonance, and relaxation [12]. The electromagnetic absorption capacity is calculated using the transmission line theory, which is shown in the equation below:

(1) $$Z_{in}=Z_{0\;}\surd \mu_{r}\;/\varepsilon_{r}\;\tan\;h\;[j(2\pi fd/c\;\surd \varepsilon_{r}/\mu_{r})]$$

(2) $$R_{L}=20\;log\;{\left|\frac{Z_{in}-Z_{0}}{Z_{in}+Z_{0}}\right|}_{\;}$$

Hence, *ε_r_* and *μ_r_* are the relative complex permittivity and permeability, respectively, *c* is the velocity of light, *f* is the microwave frequency, *d* is the thickness of the absorber, Z*_in_* is the input impedance of the absorber, and Z_0_ is the impedance of the free space [13].

### 1.3. Applications of the Microwave-Assisted Synthesis 

Microwave-assisted synthesis is used in multiple areas of chemistry for its efficiency and increased productivity [1]. The application of microwaves in organic, material, and pharmaceutical chemistry is exemplified by many microwave-assisted chemical reactions, including the Kindler thioamide synthesis, Suzuki cross-coupling reaction, Heck coupling, and Negishi coupling reaction, Diels–Alder cycloaddition reaction, Biginelli multicomponent reactions, and solid phase synthesis, among others [14]. In addition, microwave irradiation has numerous applications in the development of pharmaceutical formulations, such as drug extraction, preparation of carbon nanotubes, hydrogel formulation, solid dispersion, nanomatrix, microsphere, polymer nanoparticles, polymer gel beads, semisolid preparation, tablet coating and the drying of granules [5]. Microwave irradiation is also used in biomedicine, biotechnology, and microbiology, as well as drying compounds, food production, sterilization, and hydrodistillation [4,15]. 

### 1.4. Carbon Nanotubes (CNTs) 

CNTs were first prepared by Iijima in 1991, and subsequently, numerous research articles have been published about the preparation and modification of CNTs and their applications in nanomedicine [16]. Compared to other available nanocarriers, CNTs are highly effective molecules for drug delivery, due to their unique physical, chemical, and biological properties [17]. CNTs have a high electrical activity, large surface area, and an inherent capacity for chemical interactions with linkers and ligands through hydrophobic, π-π stacking, and hydrogen bonding interactions [18]. These characteristics allow for controlled release, high drug loading capacity, and targeting effect in tissues and organs [19,20,21]. CNTs consist of carbon-based graphene sheets rolled into a cylindrical shape and are classified according to the number of graphene wall sheets. For example, a single layer graphene sheet is known as a single-walled carbon nanotube (SWCNT), and multiple layers of graphene sheets rolled together by van der Waals (vdW forces) are known as MWCNTs [22]. The MWCNTs are typically preferable to SWCNTs because MWCNTs have (1) multiple sites for functionalization; (2) excellent stability; (3) greater rigidity that allows for a high drug loading capacity, and (4) lower probability of drug leakage [23,24]. Pristine MWCNTs (raw MWCNTs), have a low aqueous solubility due to their minimal polar surface area, poor interaction with biological molecules, low bioavailability, and accumulation in the liver [25]. These challenges could be overcome through the functionalization of MWCNTs, which increases their bioavailability and aqueous solubility [26]. However, MWCNTs are generally modified by carboxylation, esterification, amination, or cycloaddition, by covalent or non-covalent methods [27]. The covalent functionalization method consists of sidewall functionalization or ‘end and defects’ functionalization. The sidewall covalent functionalization involves the modification of sp^2^ hybridized carbon to sp^3^ hybridized carbon by the 1,3-dipolar cycloaddition reaction [18]. The best example for ‘end and defects’ covalent functionalization is the carboxylation of MWCNTs carried in strong acidic conditions [28,29]. The non-covalent methods include self-assembling modification, using biopolymers and surfactants for π-π stacking interactions and electrostatic interactions, without affecting the sidewall functionalization of MWCNTs [23,30]. Microwave irradiation is widely used for the covalent and non-covalent modification of MWCNTs because it is easy, simple, effective, not harmful to the environment and requires less time for completion of the functionalization and provides a high yield, compared to conventional methods [2]. Furthermore, the conventional methods require a large amount of chemicals or solvents, longer times for completion of the reaction and the use of conduction from thermal or electric sources [31]. Microwave methods use dielectric polarization through electromagnetic waves, which directly heats the reaction mixture and thus, the method is highly preferred for the modification and development of molecules [5]. 

MWCNTs are mainly used as carriers for the delivery of drugs and genes for the treatment of diseases. In fact, drugs are encapsulated or conjugated with MWCNTs for two main reasons: (1) for the selective delivery of drugs to the targeted sites and (2) for the sustained release of a drug into the cells [32]. There are numerous reports of loaded or conjugated drug nanocarrier MWCNTs for targeted drug delivery and sustained release, resulting in the effective inhibition of tumor growths. MWCNTs can also deliver biomolecules (e.g., gene, DNA, and small interfering RNAs (siRNAs)) and this makes them useful for gene silencing and gene delivery [33]. MWCNTs can be used as biosensors, i.e., the detection or recognition of biomolecules because MWCNTs have a large surface area and excellent electrochemical and electrical properties. Furthermore, MWCNTs can be used to detect biological organisms at low concentrations [34]. MWCNT-based biosensors have a high sensitivity to another biosensor, due to their high surface ratio and hollow tube form. MWCNTs can provide a high biological activity as they (1) immobilize enzymes; (2) produce a fast response time due to facilitation of electron-transfer reactions; (3) produce a lower surface contamination; (4) have a low redox reaction potential and (5) a longer span of existence and high stability [35]. The main aim of this review is to highlight the pharmaceutical and drug delivery applications of microwave irradiation in the design and development of functionalized MWCNTs. 

## 2. Microwave Irradiation-Based Development of MWCNTs

The microwave irradiation method is one of the most reliable methods for the modification/functionalization of MWCNTs, which occurs when different organic and inorganic nanoparticles are incorporated onto the surface of MWCNTs [23]. In this review, we discuss the preparation methods and chemical reactions recently developed for MWCNTs’ functionalized formulations using the microwave method. A number of studies have reported that the surface modification of MWCNTs with various ligands, linkers, and nanoparticles can be achieved using a single-step microwave method. This is an easy and effective process for the functionalization of MWCNTs in less time, compared to conventional methods [36]. For example, the conversion of MWCNTs into carboxylic derivatives takes 24 h and requires a large amount of highly acidic solvents. In contrast, the microwave method requires less solvent, and it only takes 10–15 min to complete the functionalization [5]. Consequently, the microwave irradiation method is now more widely used to functionalize MWCNTs. This review discusses the modification of MWCNTs using the microwave method.

### 2.1. The Carboxylation of MWCNTs Using Single-Step Microwave Irradiation Technology (MIT)

Pacheco et al. developed a novel and simple process for the oxidization of MWCNTs (2a) via the microwave irradiation of MWCNTs suspended in a sulfuric and nitric acid (1:1) mixture at 500 W for 15 cycles in 10-s intervals [37]. The results indicated that a higher degree of oxidization is achieved using microwave heating, compared to conventional heating methods [37].

**Figure 2:**
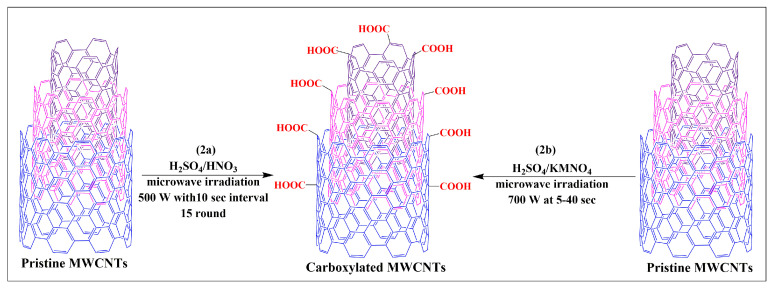
Single step synthesis of carboxylated MWCNTs through the microwave assisted method.

Pelalak and Heidari developed a novel, open-ended and shortened modified MWCNT, using a microwave-assisted method that rapidly produced modified MWCNTs in one step [38]. In this method, MWCNTs dispersed in sulfuric acid and a potassium permanganate solution were subjected to microwave heating at 700W for 5, 10, 20, 30, or 40 min (Figure 2b). Characterization studies have shown that the products obtained at 20 and 40 min were effectively functionalized MWCNTs. Dispersibility experiments indicated that functionalized MWCNTs had improved dispersibility in aqueous media without aggregation and were stable for several months, compared to pristine MWCNTs. Oxidized pristine MWCNTs were synthesized using sulfuric acid by sonication and microwave irradiation at 100 W for 10 min [39]. The oxidized pristine MWCNT functionalization significantly increased the sample/drug absorption capacity of the MWCNTs, without compromising its structure. Hojati-Talemi and Simon reported the results of three different techniques that were used to modify MWCNTs in water [40]. The first technique involved the dispersion of the MWCNTs in water after microwaving for 10 min. In the second technique, steamed water was used to disperse the MWCNTs, which were microwaved for 2 min. The third technique microwaved the MWCNTs for either 30, 60, 120, or 240 s. The results indicated that this is a simple and easy method to increase the dispersibility of MWCNTs in water, in a shorter time, compared to the conventional method for modifying MWCNTs. 

### 2.2. Functionalization of MWCNTs with Various Bioactive Molecules Using MIT

Recently, many researchers have reported the use of MIT for the functionalization of MWCNTs using amino acids, vitamins, epoxy compounds, proteins, and polymers [41,42]. Overall, MIT-based functional techniques are easy, effective, and completed in a single-step process, and they increase the magnitude of functionalization and binding in MWCNTs.

#### 2.2.1. Amino Acids and Amino Groups

**Figure 3:**
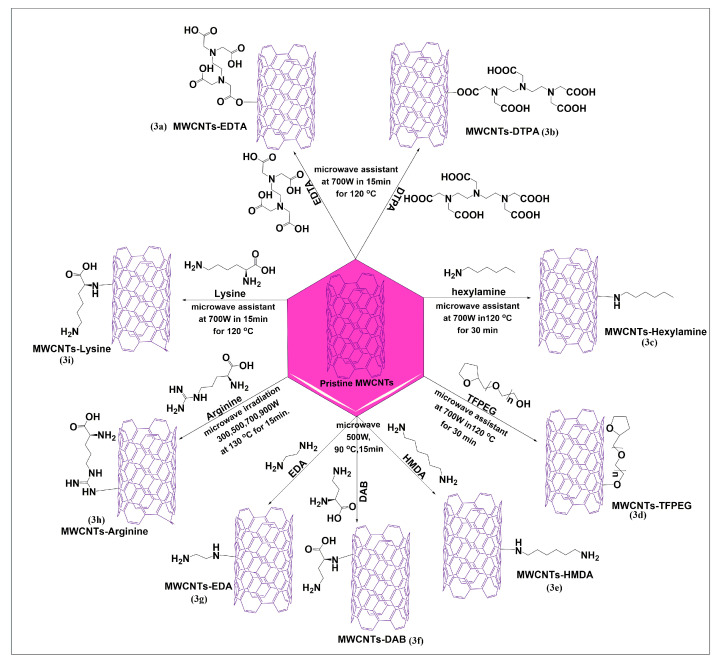
Formal synthesis of pristine MWCNTs conjugated with different types of amine linkers through microwave technology (for the sake of simplicity and clarity, multi-walled tubes are represented as single-walled tubes from here forward).

Amiri et al. reported a microwave-based Friedel–Crafts acylation reaction using two novel and effective ethylenediaminetetraacetic acid (EDTA) (Figure 3a) and diethylenetriaminepentaacetic acid (DTPA) (Figure 3b) linkers for the modification of MWCNTs, at 700 W for 15 min at 150 °C. Cadium ion adsorption studies were performed after functionalization of MWCNTs, and the results indicated that EDTA and DTPA functionalized MWCNTs highly adsorbed cadium ions, compared to pristine MWCNTs, due to negative charges on the surfaces of functionalized MWCNTs, which increased the electrostatic adsorption of cadium ions [43]. This method yielded a high magnitude of a functionalized product in less time. Figure 3c illustrates the one-pot microwave irradiation process for the functionalization of pristine MWCNTs-hexylamine, using the diazonium reaction in the presence of sodium nitrite, dimethylacetamide, and sulfuric acid [44]. In this reaction, the amino group of hexylamine reacts with the carbonyl group of MWCNTs to form covalent bonds under microwave heating at 700 W for 30 min at 120 °C. This reaction significantly increased the dispersibility of functionalized MWCNTs, compared to pristine MWCNTs in aqueous, non-acidic media. A covalent functionalization of pristine MWCNTs with tetrahydrofurfuryl polyethylene glycol, using a one-pot microwave process, was reported by Amiri et al. [45]. The pristine MWCNTs were added to aluminum chloride and tetrahydrofurfuryl polyethylene glycol, and the mixture was irradiated for 30 min at 50 °C. Hydrochloric acid was slowly added to the resulting homogenous suspension, with continuous sonication, and the mixture was irradiated at 700 W for 30 min up to 120 °C, using an industrial microwave oven. In this reaction, the hydroxyl group of tetrahydrofurfuryl polyethylene glycol reacted with the carbon atoms in MWCNTs to yield the required product (Figure 3d), which had increased solubility and dispersibility in an aqueous medium, compared to pristine MWCNTs. Three diamine compounds, 1,6-hexamethylenediamine (HMDA) (Figure 3e), 1,4-diaminobenzene (DAB) (Figure 3f), and ethylenediamine (EDA) (Figure 3g), were conjugated with pristine MWCNTs, in the presence of sodium nitrite using microwave irradiation up to 500W and heating up to 90 °C for 15 min in a one-pot method. The aqueous dispersibility of the three diamines was significantly greater than that of the pristine MWCNTs [46]. Ghiadi et al. [47] developed novel covalent amide surface-modified MWCNTs using arginine (Figure 3h) by a single pot microwave method. Arginine and MWCNTs were added to a mixture of dimethylacetamide, sulfuric acid, and sodium nitrite and the sonicated for 2 h. Subsequently, the mixture was microwaved up to 150 °C for 15 min at 300, 500, 700, or 900 W. The product was shown to be highly dispersible, with good functionalization at 900 W. The covalent conjugation of lysine with pristine MWCNTs was reported to yield lysinated MWCNTs (Figure 3i), after microwave irradiation (120 °C at 700 W for 15 min), in the presence of dimethylacetamide, sodium nitrite, and concentrated sulfuric acid [48]. The lysinated MWCNTs had an increased aqueous solubility, compared to pristine MWCNTs and had an antimicrobial efficacy in gram-negative bacteria, such as *Escherichia coli*, *Klebsiella pneumonia*, and *Salmonella typhimurium*, due to the positive charge of lysine molecules on the MWCNTs.

**Figure 4:**
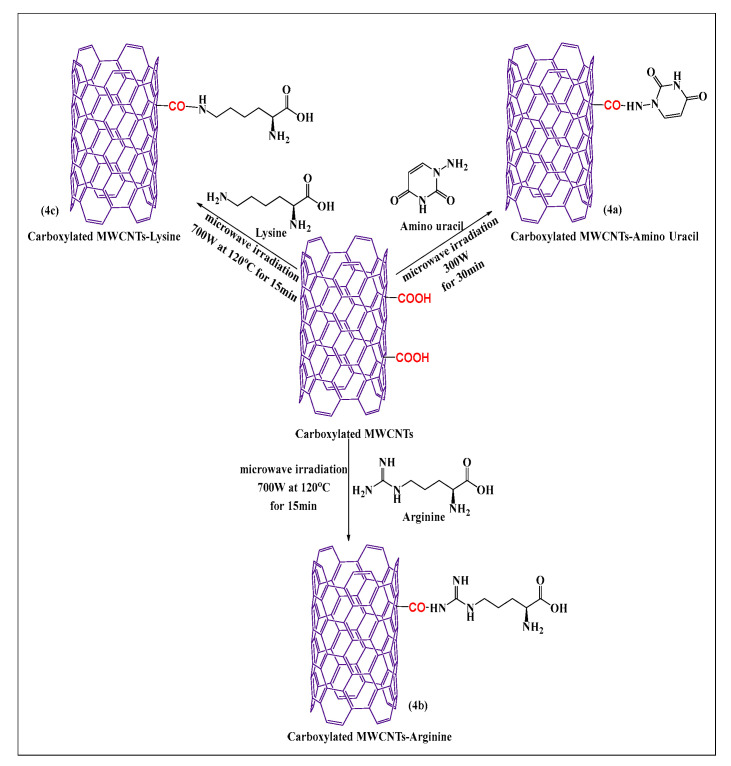
Formal synthesis of carboxylated MWCNTs reacted with an amino group under microwave irradiation.

Shahab et al. [49] designed a novel aminouracil-modified carboxylated MWCNT as seen in Figure 4a, using a microwave method. First, aminouracil was prepared in a microwave-assisted process from cyanoacetic acid and thiourea. Second, aminouracil-modified carboxylated MWCNTs were synthesized by reacting the carboxylated MWCNTs with the amine group of aminouracil at 300 W for 30 min. This process was shown to be simple and effective for producing functionalized MWCNTs that had an increased aqueous solubility, compared to carboxylated MWCNTs. Lysine and arginine conjugated MWCNTs (lysine (Figure 4b) and arginine (Figure 4c), were synthesized by the covalent conjugation of an alpha amino group of lysine and a guanidino group of arginine with carboxylated MWCNTs containing a novel nanocarrier, using a microwave reaction method at 700 W at 120 °C for 15 min [50]. These formulations had a significant in vitro efficacy in *E. coli*, *Staph. aureus*, and *S. typhimurium*, with MWCNTs-arginine producing the greatest inhibitory effect, followed by MWCNTs-lysine and pristine MWCNTs, based on data obtained from the minimum inhibitory concentration and radial diffusion assays.

#### 2.2.2. Vitamins

**Figure 5:**
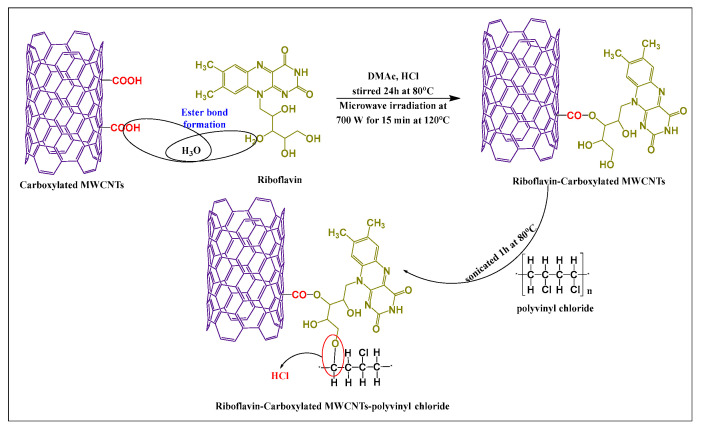
Total synthetic scheme for riboflavin modified MWCNTs by microwave irradiation.

Abdolmaleki et al. [51] synthesized vitamin-modified MWCNTs by producing covalent bonding between the hydroxyl group of riboflavin (in dimethylacetamide and concentrated hydrochloric acid) and the carboxylic group of MWCNTs, using microwave irradiation (700 W) for 15 min at 120 °C. Subsequently, the chlorine atom in polyvinyl chloride was conjugated with the free amino hydrogen of riboflavin via a complex covalent bond (Figure 5). Polyvinyl chloride was added to the riboflavin-MWCNTs suspension in the presence of tetrahydrofuran and sonicated 1 h at 80 °C. The hydrophilicity and thermal actions of the desired product were increased, compared to unmodified MWCNTs.

**Figure 6:**
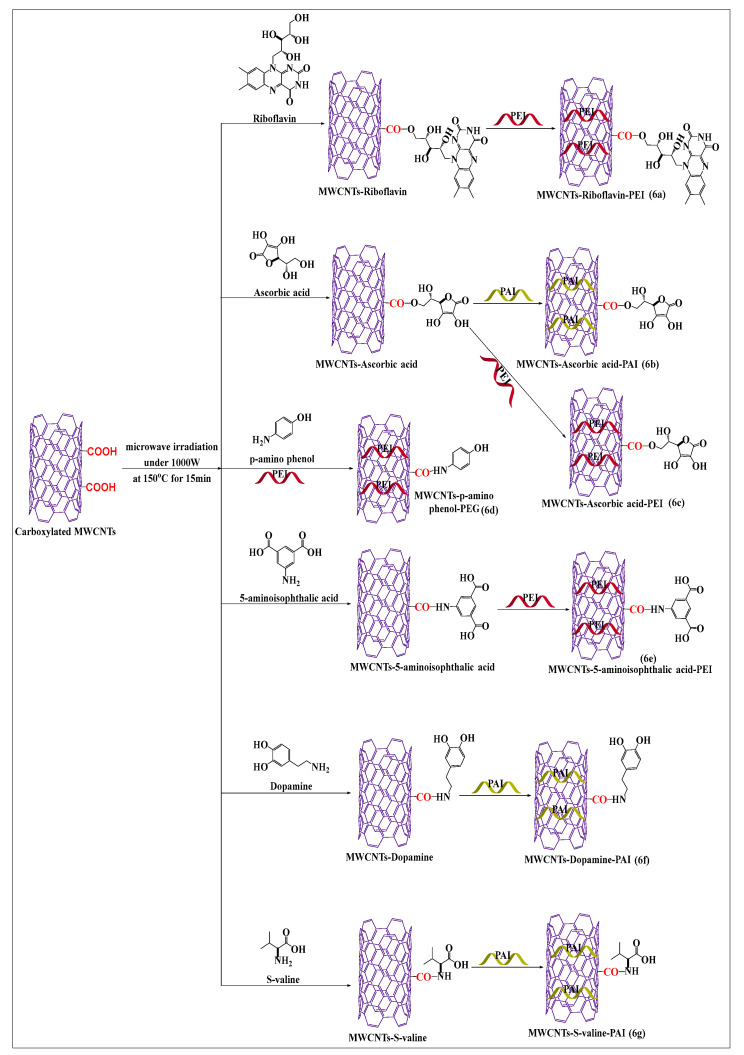
An illustration of carboxylated MWCNTs covalent conjugated with various vitamin and biomolecules using a microwave method under the same condition.

Mallakpour and Soltanian [52] designed an effective and rapid process to prepare conjugated riboflavin-MWCNTs using simple microwave-assisted techniques. Pristine MWCNTs were placed in concentrated hydrochloric acid to yield carboxylated MWCNTs, which formed ester linkages with riboflavin in the presence of dissolved dimethylacetamide, at 700 W at 120 °C for 15 min. The riboflavin-conjugated MWCNTs were incorporated into poly(ester-imide) (Figure 6a) by solution blending and sonicated for 1 h at room temperature. There was an increase in the absorption (interaction) and dispersibility of the aqueous compound, compared to carboxylated MWCNTs. Because polymers made of amino acids are typically nontoxic and can be biodegraded by tissues, they are preferred for biomedical and pharmaceutical purposes. However, no in vivo study was performed to ascertain the efficacy of the riboflavin-conjugated MWCNTs in poly(ester-imide) matrix. Mallakpour et al. [53] synthesized ascorbic acid-modified MWCNTs by polymer modifications using poly(amide-imide) (Figure 6b). The free hydroxyl group of ascorbic acid reacted with the free carboxylic group of poly(amide-imide) to form a covalent ester bond following microwave irradiation. This microwave-modified method increased the attachment, dispersibility, and interaction of MWCNTs with ascorbic acid, compared to unmodified poly(amide-imide) MWCNTs, indicating that the polymer increases the attachment of ascorbic acid. The surface modification of carboxylated MWCNTs was conducted with ascorbic acid (Figure 6c), using a microwave-assisted method (700 W for 15 min at 120 °C), followed by polyester-imide ultrasonication with polyester-imide for 1 h [53]. The covalent interactions, dispersibility, and thermal activity of the ascorbic acid-modified MWCNTs were increased, compared to unmodified CNTs. As illustrated in Figure 6d, a microwave-assisted process was used to synthesize *p*-aminophenol-conjugated carboxylated MWCNTs by forming an amide bond [54]. The formed product was placed in polyamideimide, in the presence of dimethylacetamide, for 24 h at 30–40 °C, to yield MWCNTs that had an increased dispersibility and solubility, compared to pristine MWCNTs. It was reported that oxidized MWCNTs placed in 5-aminoisophthalic acid, in the presence of microwave irradiation up to 700 W, for 15 min at 120 °C, formed an amide bond compound (Figure 6e) [55]. The resulting product had a highly stable covalent bond between the carboxylic group of MWCNTs and the free amino group of aminoisophthalic acid and a decreased the aggregation propensity, compared to unmodified MWCNTs. Mallakpour and Zadehnazari [56,57] developed two novel surface-modified MWCNT formulations by a microwave-assisted method, using dopamine and S-valine. The dopamine (Figure 6f) and S-valine (Figure 6g) compounds were suspended in dimethylacetamide and sonicated for 2 h at 60 °C. Subsequently, carboxylated MWCNTs were added to the mixture and sonicated for 2 h and microwaved for 15 min up to 700 W at 120 °C. The carboxylated MWCNTs were functionalized with polyamideimide and their solubility, dispersibility, and chemical stability were increased, compared to pristine MWCNTs.

#### 2.2.3. Proteins

**Figure 7:**
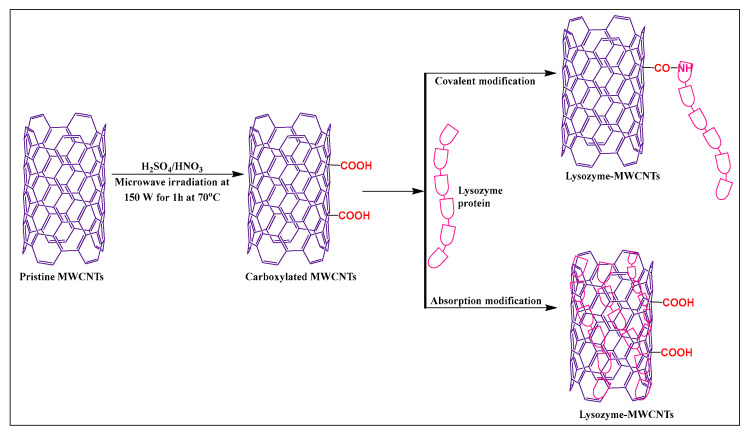
Lysozyme protein-modified with MWCNTs by covalent and non-covalent methods.

Puentes et al. [58] synthesized carboxylated MWCNTs, using sulfuric acid and nitric acid (3:1), under microwave irradiation, at 70 °C for 1 h at 150 W. The carboxylated MWCNTs were modified with lysozyme in the presence of a 2-(N-morpholino) ethanesulfonic acid (MES) buffer and N-(3-dimethylaminopropyl)-N′-ethyl carbodiimide hydrochloride, for 30 min at room temperature (Figure 7). The novel carboxylation-functionalized MWCNTs had increased stability and dispersibility in an aqueous medium compared to pristine MWCNTs.

#### 2.2.4. Epoxy 

**Figure 8:**
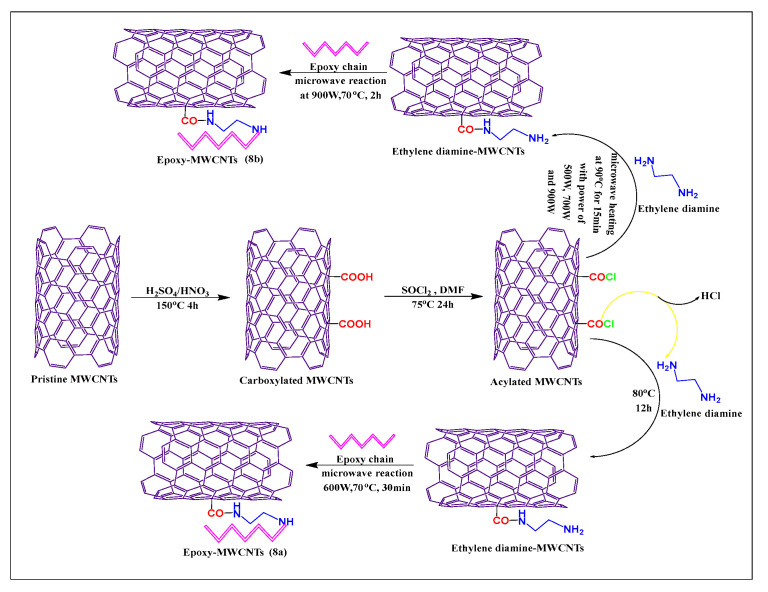
Mechanism of epoxy chain covalent conjugation with ethylenediamine-modified MWCNTs by two different methods (conventional and microwave methods).

Moaseri et al. [59] reported the synthesis of epoxy chain-functionalized MWCNTs, using a microwave method. Initially, pristine MWCNTs were oxidized with sulfuric acid and nitric acid (3:1) at 150 °C for 4 h. The oxidized MWCNTs were treated with thionyl chloride and dimethylformamide for 24 h at 75 °C, to formed acylated MWCNTs, which were then reacted with ethylenediamine and an epoxy compound, under microwave irradiation, at 600 W for 30 min at 70 °C, to yield the required product (Figure 8a). This microwave reaction increased the rate of the reaction between the epoxy chain and ethylenediamine, which increased the mechanical properties, tensile strength, and elastic modulus of the modified MWCNTs. Moaseri et al. [60] synthesized an epoxy compound using microwave-assisted precuring and the carboxylated MWCNTs were linked with ethylenediamine in the presence of sodium nitrite and sonicated for 30 min at 50 °C. 

The suspended mixture was microwaved for 15 min at 90 °C at 500, 700, or 900 W. The epoxy chain was conjugated with amine-modified MWCNTs and sonicated for 4 h at 50 °C and 900 W at 70 °C for 2 h, to yield the final product (Figure 8b). The epoxy-modified compound had a greater tensile strength when microwave-assisted precuring was used, compared to a hot plate or oven precuring methods. 

### 2.3. Covalent and Non-Covalent Modified MWCNTs Nanoparticles by MIT 

**Figure 9:**
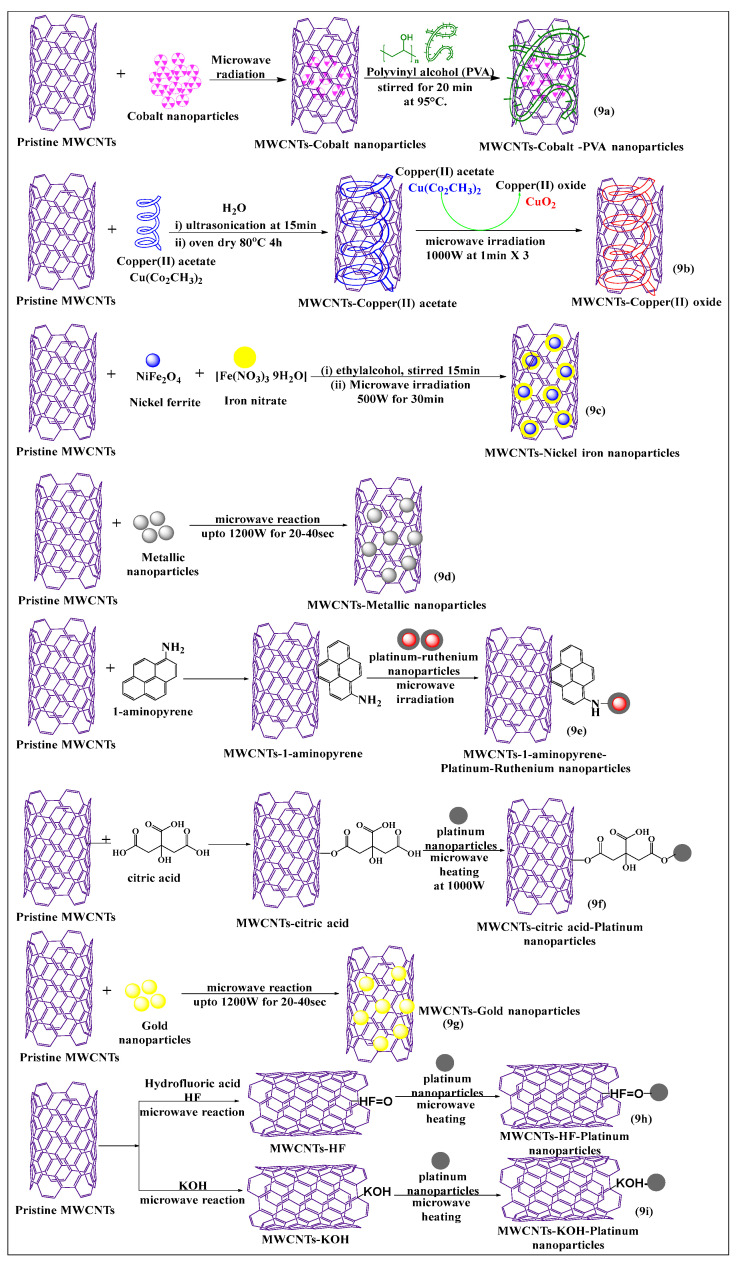
Graphical representation of pristine MWCNTs covalent and non-covalent modified with various nanoparticles using a microwave-assisted method.

Various types of nanoparticles, such as gold, silver, platinum, and polymeric, are typically used for the modification of MWCNTs by MIT, as this increases the loading capacity and solubility of the MWCNTs [61]. Elena et al. [62] synthesized non-covalently conjugated cobalt nanoparticle MWCNTs (Figure 9a), using a microwave absorption method. The surface was modified with polyvinyl alcohol and a directly applied magnetic field to increase the segregation of MWCNTs. This approach was shown to be a simple and rapid process for the preparation of cobalt-MWCNT nanoparticles. Rudd et al. [63] described the microwave-assisted synthesis of copper oxide-decorated carbon nanotubes (Figure 9b), which could be used as precursors for ultra-conductive copper wires. In this method, copper acetate was converted to copper oxide and subsequently deposited on MWCNTs, under microwave irradiation, to produce tenorite-decorated CNTs (CuO-CNTs), which had a high loading capacity and excellent dispersion, compared to non-modified MWCNTs. Rigo et al. [64] developed an effective, surface-modified nickel ferrite MWCNT (Figure 9c)-based nanocarrier, using a microwave absorption technique. In this reaction scheme, nickel ferrite and iron nitrate were dissolved in ethyl alcohol, and the resulting MWCNTs were dissolved in the same solvent and mixed properly for the formation of the suspension and stirred for 24 h at room temperature. Subsequently, the suspension was microwaved at 500 W for 30 min, and the same procedure was used to prepare a pure nickel ferrite nanocarrier for comparative studies. The nickel ferrite/multi-walled carbon nanotube (NiFe_2_O_4_/MWCNTs) composite had a higher catalytic activity for the treatment of dye-contaminated water waste than pure NiFe_2_O_4_, indicating synergism between NiFe_2_O_4_ and MWCNTs. In another study, pristine MWCNTs were decorated with metallic nanoparticles (Figure 9d) and microwaved at 1200 W for 20–40 s at 10 s intervals, yielding a novel 3D metallic MWCNT formulation. Because of the metallic coating, these MWCNTS were considered to be highly useful for electrochemical applications [65]. 

Jiang et al. [66] synthesized a novel, pristine MWCNTs formulation that was noncovalently modified, using a sonicated conventional method, and platinum-ruthenium nanomolecules were conjugated with 1-aminopyrene-modified MWCNTs (Figure 9e) and microwaved for 2 min. This functionalization process increased the structural integrity and electronic activity of the MWCNTs, compared to conventional acid-treated MWCNTs. Pristine MWCNTs were covalently functionalized with citric acid, using a simple stirring conventional method. The citric acid-modified MWCNTs were microwaved with platinum nanoparticles (Figure 9f) at 160 °C for 2 min at 1000 W. The resulting product had an increased dispersibility and electrochemical activity, compared to platinum-modified MWCNTs through a conventional reflux process [67]. Pristine MWCNTs were directly decorated with gold nanoparticles (Figure 9g), without oxidization, using a microwave (700 W for 20 s at intervals of 10 s after every rotation of the reaction), by an easy one-pot method, which had a good dispersibility in the presence of a gold nanoparticle in the MWCNTs, as compared to pure MWCNTs [68]. Shibin et al. [69] used an intermittent microwave method to functionalize pristine MWCNTs, using hydrofluoric acid (HF) (Figure 9h). A defective covalent interaction and oxidation occurred following the addition of hydrogen peroxide, and the added platinum nanoparticles interacted with the modified MWCNTs, after microwave irradiation after several cycles (5 s on/5 s off). This process yielded a product that had a greater interaction with the platinum nanoparticles, which increased the stability of MWCNTs. The MWCNTs were placed in potassium hydroxide (Figure 9i), followed by microwaving (2000 W for 15 s on/off process) and the platinum nanoparticles were incorporated on the modified MWCNTs, which had a good stability and a uniform loading of nanoparticles [70].

**Figure 10:**
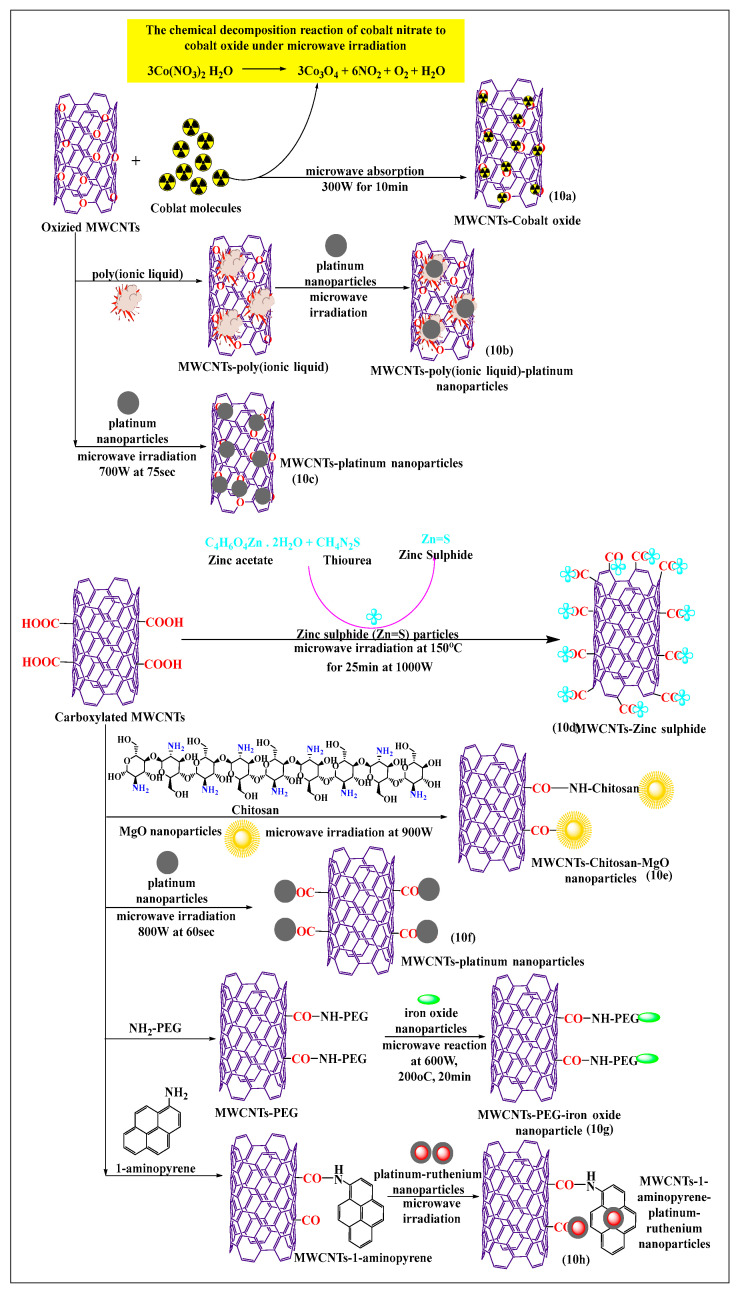
Schematic reaction of carboxylated MWCNTs conjugated with nanoparticles through microwave methods.

Bakr et al. [71] recently reported the synthesis of oxygen-functionalized MWCNTs by the reacting nitric acid under reflux conditions, with the cobalt precursor Co(NO_3_)_2_·6H_2_O, to attach the oxide of the cobalt atom on the oxygen-modified MWCNTs using a microwave. The suspension (cobalt oxide and oxygen-modified MWCNTs) was stirred for 1 h, sonicated at 45 min, and microwaved at 300 W for 10 min or 15 min at 600 W, which increased the catalytic activity of the formulation, compared to cobalt oxide. This method was a rapid and easy process for the modification of cobalt precursors, i.e., the chemical decomposition reaction of cobalt nitrate to cobalt oxide produced by microwaving (Figure 10a). Tamilarasan and Ramaprabhu [72] synthesized MWCNTs with a polyionic liquid and platinum nanoparticles (Figure 10b), using a three step process. The MWCNTs were oxidized using a chemical vapor deposition process, where the MWCNTs were placed in an acid solvent to form oxide molecules, and the oxidized MWCNTs were non-covalently modified with a polyionic liquid using a polymerization method. The platinum nanoparticles used for surface absorption on the poly(ionic liquid)-MWCNTs and pristine MWCNTs were microwaved, the poly(ionic liquid)-conjugated MWCNTs had an increased solubility and dispersibility, compared to pure MWNTs. Mezalira and Bron [73] used a microwave to synthesize MWCNTs with platinum nanoparticles to increase the stability of the MWCNTs (Figure 10c). Chloroplatinic acid was used for the synthesis of platinum nanoparticles that were loaded on MWCNTs using a microwave method, with ethylene glycol as a solvent, under sonication for 30 min, at 700 W for 75 s. The platinum-loaded MWCNTs were more stable than oxidized or pristine MWCNTs. Zinc sulfide nanoparticles were prepared by non-covalent incorporation into carboxylated MWCNTs (Figure 10d), using a microwave method, which increased the thermal stability [74]. In this process, zinc acetate reacted with thiourea to yield zinc sulfide, which was incorporated into the carboxylated MWCNTs after dispersion in water and ultrasonication for 15 min, followed by microwaving at 2.45 GHz for 15 min at 150 °C. Shariatzadeh and Moradi [75] synthesized chitosan and magnesium oxide nanoparticles for loading on to carboxylated MWCNTs (Figure 10e) using a single-step microwave-assisted process. Chitosan, magnesium oxide, and carboxylated MWCNTs were mixed and placed in a microwave oven for 30 min. This allowed the free amino group of chitosan to react with the free carboxylic group of MWCNTs to form an amide bond, and the remaining free carboxylic groups reacted with the magnesium oxide nanoparticles and with the hydroxyl group of chitosan. 

There was a strong interaction of chitosan and magnesium oxide nanoparticles in the MWCNTs, compared to conventionally treated MWCNTs. Serban et al. [76] used a surface modification method to oxidize MWCNTs with platinum (Figure 10f) using a microwave-assisted technique. The MWCNTs were oxidized with sulfuric acid and nitric acid under reflux conditions, and the oxidized MWCNTs were incubated with chloroplatinic acid to modify the surface in the presence of ethylene glycol under microwave heating up to 800 W for 60 s. The functionalized MWCNTs, decorated platinum had an increased stability, compared to pristine MWCNTs decorated with platinum. In MWCNTs modified with polyethylene glycol (Figure 10g), pristine MWCNTs were oxidized in the presence of sulfuric acid and nitric acid, using microwave heating at 1000 W, over a range of 200 °C to 220 °C for 20 min [77]. The carboxylated MWCNTs were reacted with polyethylene glycol in a microwave for 30 min, followed by a reaction with iron oxide nanoparticles at 600 W at 200 °C for 20 min. The resulting composite catalyst could be used for oxidative degradation. This novel poly-formulated MWCNT used effluent and wastewater treatment, as the dissolved ions removed and neutralized the acidic compounds. It was reported that carboxylated MWCNTs noncovalently modified with 1-aminopyrene and reacted with platinum-ruthenium nanoparticles (Figure 10h), when microwaved for 2 min, increased the electrochemical activity and loading capacity of platinum-ruthenium nanoparticles, compared to conventional acid-treated MWCNTs [78]. 

### 2.4. Graphene and Other Polymers Used for the Decoration of MWCNTs

**Figure 11:**
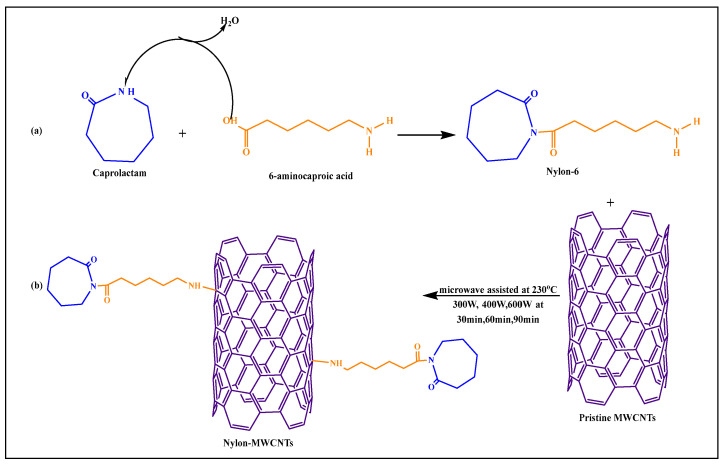
(**a**) Synthesis of Nylon-6, (**b**) Reaction scheme for synthesis of nylon-6 functionalized MWCNTs through microwave technology.

Macias et al. [79] formulated a pristine MWCNT-nylon-6-conjugated covalent product using microwave-based polymerization methods. Nylon-6 was synthesized by the reaction of 6-aminocaproic acid with caprolactam, where the carboxylic acid group of 6-aminocaproic acid reacted with the amino group of caprolactam to form an amide bond, yielding nylon-6 (Figure 11), by a microwave-assisted process. The amine group of nylon-6 reacted with pristine MWCNTs after microwaving at 300, 400, or 600 W for 30, 60, or 90 min, respectively, at 230 °C. The conjugated products, such as nylon-6, pristine MWCNTs, and nylon-conjugated MWCNTs, had a higher dispersibility and compatibility, compared to pure nylon-6 nanoparticles. 

**Figure 12:**
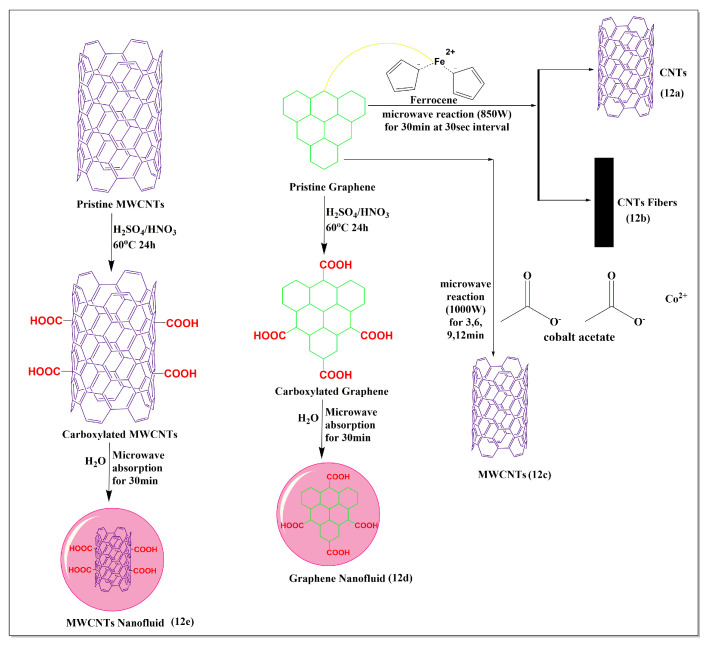
Illustration of the use of graphene for the modification of MWCNTs under microwave conditions.

Maria et al. [80] reported a clean and sustainable ultrafast method for the synthesis of carbon nanotubes (Figure 12a) and carbon fibers (Figure 12b), embedded with magnetic nanoparticles (Fe_3_O_4_) by microwave irradiation of a mixture of ferrocene and graphite at 850W. The microwave method used for the preparation of nanotubes provided a more sustainable low-cost method with high yields, compared to other conventional methods, such as thermal decomposition, arc discharge, chemical vapor deposition, and laser ablation, which are widely used for the preparation of carbon nanotubes [80]. Cervantez et al. [81] synthesized efficient sequestering MWCNTs (Figure 12c) from a mixture of graphite and cobalt acetate powders using a microwave (1000 W at 30 s intervals for reaction times of 3, 6, 9, and 12 min). The sequestration was dependent on the length of the microwave heating, which yielded maximum sequestration after 12 min, producing MWCNTs with diameters of 20–50 nm. Karami et al. [82] synthesized a MWCNT (Figure 12e) and graphene nanofluid (Figure 12d), using a microwave method. The carboxylated MWCNTs were synthesized by stirring graphene, sulfuric acid, and nitric acid (3:1), at 60 °C for 24 h. Subsequently, deionized water was added (0.1% or 0.2%) to the mixture containing carboxylated MWCNTs and graphene, followed by microwaving for 30 min. The viscosity and shear stress experiments indicated that carboxylated graphene had a lower viscosity and shear stress, compared to carboxylated MWCNTs. There was an increase in the concentration and temperature and a decrease in the surface tension and density of the MWCNTs and graphene nanofluids, compared to deionized water.

### 2.5. Miscellaneous Chemical Molecules Used to Modify MWCNTs

**Figure 13:**
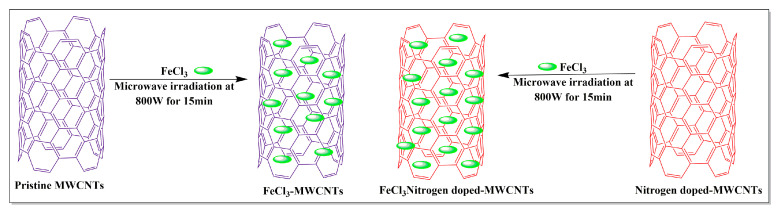
Iron chloride loaded with MWCNTs by the microwave-assisted method.

Several compounds, including, but not limited to, ferric chloride, 2,3-diaminopyridine, imidazole and polyamide-6, have been used in the synthesis of MWCNTs. Kumar et al. [83] conducted a comparative study of pristine MWCNTs and nitrogen-doped (i.e., MWCNTs with nitrogen introduced into the graphene lattice) MWCNTs. These nanotubes were synthesized separately using a chemical vapor deposition method where the ferric chloride nanoparticles (FeCl_3_) were added to pristine MWCNTs and the MWCNTs were exposed to nitrogen, using a sonication method and microwaved at 800 W for 1.5 min (Figure 13). The results indicated that the nitrogen-exposed MWCNTs had a high magnetic-inducing capacity and a high metal oxide loading capacity, compared to pristine MWCNTs.

**Figure 14:**
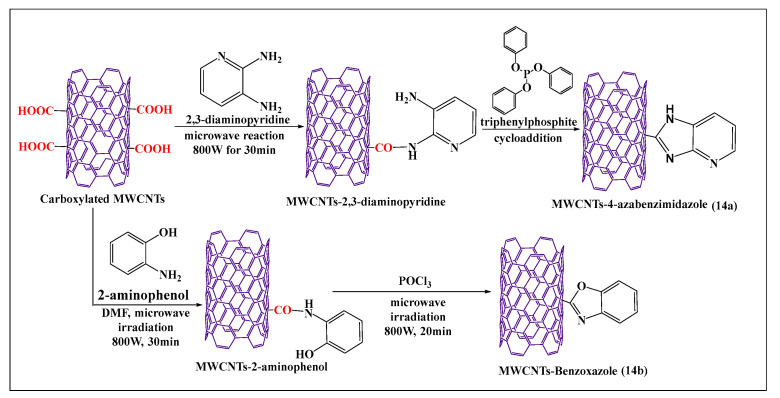
Schematic presentation of the cycloaddition modification of carboxylated MWCNTs.

Azizian et al. [84] reported the amination and cycloaddition of MWCNTs (Figure 14a), using a microwave method, where the amine group of 2,3-diaminopyridine reacted with a carboxylic group of MWCNTs in the presence of dimethylformamide and triphenylphosphate after microwaving the mixture for 30 min. This approach was shown to be a simple and short process for the functionalization of MWCNTs, compared to the conventional process, and the product had a higher level of aqueous solubility, compared to pristine MWCNTs. Tahermansouri et al. [85] reported a simple and effective one step microwave process for the functionalization of oxidized MWCNTs with 2-aminophenol (Figure 14b), at 800 W for 30 min. The aminated MWCNTs were placed in phosphoryl trichloride and microwaved at 800W for 20 min, yielding benzoxazole-MWCNTs, which were more soluble in dimethylformamide than nonfunctionalized MWCNTs. Oxidized pristine MWCNTs were synthesized by the sonication of pristine MWCNTs with nitric acid and sonicated for 40 min, followed by microwaving for 30 min, to induce the oxidizing reaction [86]. 

**Figure 15:**
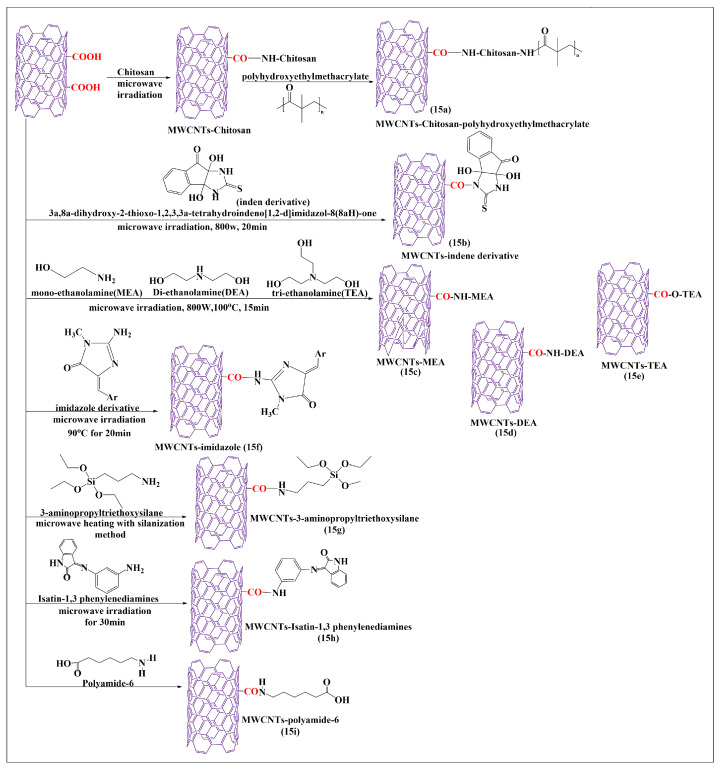
Total synthesis of MWCNTs conjugated with various molecules by the microwave-assisted method.

The oxidized MWCNTs were conjugated with chitosan after an additional 30 min of microwaving (i.e., the carboxylic group of MWCNTs reacted with the free amine group of chitosan to form an amide bond) and mixed with poly-2-hydroxyethylmethacrylate. The free amine group in the chitosan reacted with the carboxylic group of the poly-2-hydroxyethylmethacrylate polymers in the presence of pyridine to form covalent bonds (Figure 15a). The characteristic analysis indicated that the prepared formulations had a higher dispersibility in an aqueous medium, compared to pure MWCNTs, which increased the volume of conjugation for chitosan and poly-2-hydroxyethylmethacrylate in the oxidized MWCNTs. Tahermansouri and Abedi [87] conducted a comparative study with thermal and microwave methods for the functionalization of short MWCNTs, with the indene derivative 3a,8a-dihydroxy-2-thioxo-1,3,3a,8a-tetrahydroindeno [1,2-d]imidazol-8(2H)one (Figure 15b). In this study, thiourea and ninhydrin were used to synthesize an indene derivative that was conjugated with oxidized MWCNTs, in the presence of dimethylformamide. The microwave method was shown to be a simple and rapid process (25 min at 800 W), as compared to the thermal methods (96 h at a temperature to 95 °C). Cell proliferation experiments with indene-functionalized MWCNTs and oxidized MWCNTs in MCF-7 (breast cancer) or MKN-45 (gastric cancer) cells, indicated that indene-functionalized MWCNTs showed a higher cytotoxicity in MKN-45 cells (77%) and MCF-7 cells (65.6%), as compared to oxidized MWCNTs in MKN-45 cells (72.2%) and MCF-7 cells (47.2%). Furthermore, the dispersibility of MWCNTs in an aqueous medium improved after functionalization of indene with MWCNTs. Davarpanah et al. [88] developed esterified and aminated MWCNTs using a microwave-assisted process with monoethanolamine (Figure 15c), diethanolamine (Figure 15d), and triethanolamine (Figure 15e). First, pristine MWCNTs were oxidized in the presence of sulfuric and nitric acid, with continuous stirring, for 12 h at room temperature. Second, the oxidized MWCNTs were conjugated with either monoethanolamine, diethanolamine, or triethanolamine, with sodium nitrate and dimethylformamide, under sonication for 10 min and were microwaved at 110 °C for 15 min up to 700 W. The carboxylic group of the oxidized MWCNTs reacted with an amino group in monoethanolamine, diethanolamine, and triethanolamine to form an amide bond. Finally, the oxidized MWCNTs were converted to acylated MWCNTs after 12 h of stirring with thionyl chloride. The acylated MWCNTs reacted with the ethyl group of mono-ethanolamine, diethanolamine, or triethanolamine to form a covalent bond by an electrophilic addition reaction. The acylated MWCNTs were mixed with dimethylformamide and either monoethanolamine, diethanolamine, or triethanolamine, and sonicated for 10 min and microwaved at 700W for 15 min at 110 °C. The final step conjugated ethylenediamine and the acylated MWCNTs for comparative experiments, which indicated that the monoethanolamine, diethanolamine, and triethanolamine-modified MWCNTs had a higher dispersion in an aqueous medium, compared to ethylenediamine-modified MWCNTs. Zardini et al. [89] reported the conjugation of MWCNTs with ethanolamine using a microwave synthesis technique. The oxidized pristine MWCNTs (Figure 15c–e) were placed in sulfuric and nitric acid for 12 h at room temperature. The oxidized MWCNTs were conjugated with either mono-, di-, and tri-ethanolamines, in the presence of dimethylformamide, and sonicated for 10 min until a suspension was formed and was microwaved for 15 min up to 800 W at 100 °C. The MWCNTs functionalized with triethanolamine had a slightly greater antimicrobial efficacy in gram-positive bacteria, compared to di- and monoethanolamine, respectively. A covalent modification of MWCNTs was reported using novel imidazole derivatives (Figure 15f) and a microwave method [90]. The MWCNTs were conjugated with creatinine by mixing MWCNTs and creatinine in the presence of dimethylformamide, and sonicated for 15 min and the suspension was microwaved for 20 min at 800 W. The amide-modified MWCNTs reacted with imidazole derivatives after microwaving the mixture at 800 W for 20 min. The composite amide-modified MWCNTs decreased the proliferation of cancer cells and had a greater efficacy in gastric (MKN-45), compared to breast (MCF-7) cancer cells, the % of the cell viability was found to be 76.5% and 56.3%, respectively.

Bonalume et al. [91] conducted a comparative analysis of MWCNTs functionalized by carboxylation and silanization using either a microwave or a conventional process. The microwave process led to the reaction of MWCNTs with sulfuric and nitric acid at different reaction times and powers (150 W or 300 W, at 10, 20, or 30 min). In the conventional thermal process, MWCNTs also reacted with the same compounds, but required a temperature of 50 °C for 9 h. The microwave and conventional methods used 3-aminopropyltriethoxysilane to form silanizated MWCNTs (Figure 15g), which can interact with organic and inorganic compounds. Compared to the conventional process, the microwave process decreased the reaction time, and a lower amount of acid was required for the synthesis of the MWCNTs. Zomorodbakhsh et al. [92] synthesized isatin-conjugated MWCNTs, using 1,3 phenylenediamines and isatin, to form isatin-3-arylimines MWCNTs (Figure 15h) using a microwave method. In this reaction, the amino group of isatin-3-arylimines reacts with the carboxylic group of MWCNTs. This microwave synthesis process was rapid and effective for producing functionalized MWCNTs that had an increased solubility in an aqueous medium, compared to the conventional acid treatment. Huang et al. [93] synthesized novel polyamide-6-grafted MWCNTs (Figure 15i) using a microwave. Polyamide-6 reacted with the carboxylic groups of MWCNTs to form covalent bonds after microwaving the mixture at 200 °C for 4 min. The resulting compounds were shown to have an increased grafting capacity, dispersibility, and interfacial interaction, compared to carboxylated MWCNTs.

### 2.6. MWCNTs for Drug Delivery

The use of MWCNTs for drug and gene delivery for various drug classes, such as chemotherapeutics and photodynamic therapy, have been extensively reviewed [94]. Due to the surface functional groups, the MWCNTs can increase the “enhanced permeability and retention” (EPR) effect in tumors, thus enabling the targeted delivery to various cancer tumors. Interestingly, MWCNTs without drug cargo have been reported to inhibit tumor growth [95].

Li et al. [92] used RGD-decorated chitosan (CS)-functionalized single-walled carbon nanotube (SWCNT) to load and deliver docetaxel (DTX). The DTX-loaded RGD peptide and chitosan-functionalized carbon nanotubes (CNTs) released a high amount of DTX at pH 5.0 (68%) and a low amount at pH 7.4 (49%), showing that the CNT formulations offered sustained release at the tumor site. Receptor-ligand targeted delivery of docetaxel at tumor sites increased the antiproliferative efficacy of DTX (IC_50_ = 4.11 µg/mL) in A549 lung cancer cells, compared to pure docetaxel (IC_50_ = 56.92 µg/mL). However, DTX-loaded RGD peptide and chitosan functionalized CNT formulations had a lower efficacy in MCF-7 cancer cells, compared to alone DTX. In vivo experiments in A549 tumor-bearing nude mice indicated that DTX-loaded RGD peptide and chitosan-functionalized CNT formulations significantly decreased tumor growth, compared to pure DTX, by 79 and 34.5%, respectively. Overall, these in vitro and in vivo results indicated that functionalized CNTs are an effective nanocarrier for delivering DTX to A549 cancer cells and tumors [96]. MWCNTs functionalized with polyethylene-glycol (PEG) and loaded with ABT-737 (an inhibitor of the anti-apoptotic proteins, Bcl-XL, Bcl-2 and Bcl-w; [97]) for mitochondrial targeting in the A549 cancer cell. The in vitro proliferation and apoptosis experiments indicated that ABT-737-loaded PEG-functionalized MWCNTs elicited a higher cytotoxicity, compared to pure ABT-737, and drug free functionalized MWCNTs showed a negligible cytotoxicity in A549 cancer cells. ABT737-loaded PEGylated CNTs showed anincreased cancer cell uptake and mitochondrial accumulation via macropinocytosis and clathrin-mediated endocytosis. The cytosolic release of the drug from CNTS produced apoptosis in the cancer cells, and there was a significant and rapid decrease in the mitochondrial membrane potential and the generation of the intracellular reactive oxygen species. The findings indicate that PEGylated MWCNTs formulations are non-toxic, effective nanocarriers for drug delivery of ABT-737 to A549 cancer cells [97]. MWCNTs conjugated with cyclic arginylglycylaspartic acid (RGD) peptide were used for the targeted delivery of camptothecin to integrin αvβ3-expressing A375 human cancer cells. In A375 cells, the RGD peptide-conjugated MWCNTs was more than 3-fold more efficacious than the RGD peptide free MWCNTs in inhibiting the proliferation of A375 cells. In contrast, neither formulation significantly inhibited MCF-7 cell proliferation, as these cells do not express αvβ3 integrin. The uptake of RGD peptide linked MWCNTs was greater than in the A375 cells and they also had increased aqueous dispersibility, making them an excellent nanocarrier for the targeted delivery of anticancer drugs to αvβ3-expressing cancer cells [98]. Hydroxypropyl-β-cyclodextrin (HP-β-CD)-functionalized single-walled CNTs were prepared and loaded with the anticancer drug formononetin (FMN). The HP-β-CD-functionalized SWCNTs showed an enhanced dispersibility in an aqueous medium, compared to unmodified CNTs. The FMN loading efficiency in functionalized SWCNTs was 88.66%, and the drug release kinetics demonstrated a slow and sustained release. The cell cytotoxicity (IC_50_) of HP-β-CD-functionalized SWCNTs loaded with formononetin and pure formononetin inhibited the proliferation of MCF-7 (IC_50_ values of 17.989 ± 1.255 and 55.986 ± 2.479 μmol/L, respectively) and Hela cells (21.775 ± 1.338 and 72.995 ± 0.551 μmol/L, respectively), indicating that the FMN-loaded HP-β-CD-functionalized SWCNTs enhanced the cell cytotoxicity in both MCF-7 and Hela cells, compared to pure formononetin [99]. Mangiferin-loaded polyethylene glycol-linked carbon nanotubes (CNTs) were 1.28-fold more efficacious in U-87 brain cancer cells, compared to pure mangiferin. Mechanistic studies indicated that functionalized MWCNT formulations produce a greater apoptotic rate (54.78%), compared to pure mangiferin (20.63%), due to the increased cellular uptake and permeability, and the IC_50_ values were 162.91 µM and 208.48 µM, respectively. The bioavailability and retention duration of mangiferin were increased in in vivo pharmacokinetic studies in male Wistar rats. The results demonstrate that PEGylated MWCNTs are effective nanocarriers for the phytochemical delivery and bioavailability enhancement [100]. Docetaxel-loaded piperine-conjugated MWCNTs significantly decreased the proliferation of MCF-7 and MDA-MB-231 breast cancer cells (IC_50_ values of 8 μg/mL and 6 μg/mL, respectively), compared to pure docetaxel (IC_50_ values of 25 μg/mL and 15 μg/mL, respectively). The AUC for docetaxel-loaded, piperine-conjugated MWCNTs was 6.4 times that of docetaxel alone after a single dose of 5 mg/kg in male Wistar rats. These piperine-conjugated MWCNTs had increased anticancer efficacy and this formulation could be used as an effective nanocarrier for the docetaxel delivery in the treatment of breast cancer [101]. Hyaluronic acid-functionalized CNTs loaded with doxorubicin have show to target breast cancer cells [102]. In vitro, doxorubicin-loaded functionalized CNTs and pure doxorubicin in inhibited the proliferation of MDA-MB-231 breast cancer cells by 37.72 ± 1.03% and 73.45 ± 1.54%, respectively. Cellular uptake of doxorubicin-loaded functionalized CNTs in MDA-MB-231 was 2.4-fold greater than pure doxorubicin. The results indicate that the prepared formulations were more efficacious in inhibiting the proliferation of breast cancer than pure doxorubicin because hyaluronic acid coupled CNTs allow for the targeted delivery of drugs to the tumor site. Water insoluble anti-cancer drugs have been shown to be successfully delivered to cancer cells, using CNTs loaded with anticancer drugs, such as paclitaxel [103]. SWCNTs that have been noncovalently functionalized with chitosan are grafted with hyaluronic acid to fabricate biocompatible nanocarriers for paclitaxel delivery to A549 cells. The resulting SWNTs–CHI–HA nanotubes displayed a significant drug loading capacity and cytotoxicity against A549 cells, while having no toxic effects on normal cells, thereby establishing these SWCNTs as effective for delivering cancer chemotherapeutics. The co-loading and release of doxorubicin and paclitaxel from pristine SWCNT, chitosan-functionalized SWCNT and protonated f-SWCNT, were investigated using all-atom molecular dynamic (MD) simulations [104]. The results indicated that the co-loading of DOX and PTX to the pristine SWCNT is exothermic and spontaneous. The DOX molecules predominantly interact with the SWCNT via π–π stacking through the conjugated aromatic rings, while the separated aromatic rings of the PTX also primarily interacted with the SWCNT, through π-π stacking yet supplemented by X-π (X=C-H, N-H and C=O). Non-covalent functionalization of chitosan decreased the interaction of both drugs with SWNTs, while protonation of chitosan further inhibited the association of Dox and PTX, facilitating the drug release from the sidewalls.

Many studies have used folic acid (FA)-conjugated MWCNTs for the delivery of anticancer drugs. MWCNTs functionalized with folic acid, via a PEG linker, were used to load doxorubicin by non-covalent interactions, and then cisplatin was encapsulated within the modified MWCNTs, to formulate a dual-drug delivery system with pH-sensitive release properties for treating breast cancer [105]. The dual-drug release from functionalized MWCNTs was 22% and 26% for doxorubicin and cisplatin, respectively, at pH 6.5, but only 8% and 13% of doxorubicin and cisplatin, respectively, were released at pH 7.4 after 72 h. In vitro efficacy experiments indicated that doxorubicin and cisplatin-loaded MWCNTs functionalized with folic acid decreased cell viability by 20% in MCF-7 breast cancer cells, at pH 6.5, compared to a 40% decrease in cell viability at pH 7.4. This data clearly indicated that the proposed formulation provided a pH-sensitive efficacy, which is critical for targeting cancerous tumors as they are typically in an acidic environment. Amino-functionalized MWCNTs were linked concomitantly to four different functional moieties (fluorochrome Af-488/647, folic acid (FA), methotrexate (MTX), and radiotracer (99mTc) to construct FA-MTX co-tethered MWCNTs as a novel class of theranostic prodrug. The formulated MWCNTs had site specific and a selective antitumor efficacy in in vitro and in vivo models [106]. The IC_50_ values of the formulated MWCNTs to inhibit in MCF-7 and A549 cancer cells were 1.95 and 2.13 µM, respectively. In contrast, the IC_50_ values for pure MTX were 7.36 and 7.3 µM respectively. In vivo tumor accumulation, antitumor efficacy and toxicity experiments indicated that the MWCNTs selectively accumulated in the cancer cells, had a greater antitumor efficacy than pure MTX, and did not produce overt toxicity. Docetaxel-loaded chitosan-folic acid grafted MWCNTs have been reported to have a significant efficacy and decreased toxicity in lung cancer cells, compared to pristine MWCNTs [107]. MWCNTs modified with chitosan-folic acid had a higher docetaxel loading capacity than unfunctionalized MWCNTs, with loading efficiencies of 80% and 60%, respectively. In A549 cells, the efficacy of docetaxel-loaded chitosan-folic acid-grafted MWCNTs was greater (IC_50_ = 0.56 µg/mL) than docetaxel alone (IC_50_ value 50.19 µg/mL). The presence of folic acid increased the antitumor efficacy of the MWCNTs because folic acid can interact with the folate receptor, which is overexpressed in A549 lung cancer cells [107].

Folic acid-PEG bis-amine functionalized carboxylated MWCNTs delivered 5-fluorouracil effectively and sustainably, based on in vitro and in vivo data obtained in breast cancer [108]. The cellular uptake of 5-fluorouracil-loaded MWCNTs was 2-fold greater than 5-fluorouracil alone in MCF-7 cells, indicating that 5-fluorouracil loaded MWCNTs increased the targeted delivery of 5-fluorouracil to breast cancer cells, resulting in greater efficacy in MCF-7 cells, compared to pure 5-fluorouracil. Thiamine and riboflavin functionalized MWCNTs encapsulated with paclitaxel effectively delivered paclitaxel to breast cancer cells and increased the dispersibility of the MWCNTs [109]. Paclitaxel-loaded ligand functionalized MWCNTs were significnatly more efficacious than pure paclitaxel in inhibiting the proliferation of MCF-7 cells, due to improved targeting and increased cellular uptake due to the surface conjugation with the targeting ligands, thiamine and riboflavin. The anticancer efficacy of MWCNTs coated or covalently conjugated with D-alpha-tocopheryl polyethylene glycol 1000 succinate (TPGS) and encapsulated with docetaxel, was compared to the *i.v.* administration of docetaxel (DocelTM). The results indicated that TPGS conjugated MWCNT were more efficacious and safer than uncoated or TPGS coated MWCNT [110]. MWCNTs grafted with the copolymer (poly(acrylic acid) and polyethylene glycol) were shown to be optimal nanocarriers for the delivery of cyclophosphamide and methotrexate [111]. Doxorubicin-loaded multi-walled carbon nanotubes (MWCNTs-TC) with transactivator of transcription (TAT)-chitosan functionality were taken up by BEL-7402 hepatoma cells where the drug was released at an intratumoral pH, producing an antitumoral activity in vitro and in vivo [112]. The doxorubicin-loaded TAT-chitosan-functionalized MWCNTs were more efficacious than pure doxorubicin in inhibiting the proliferation of BEL-7402 cells. Furthermore, in an in vivo xenograft rodent model, the doxorubicin-loaded TAT-chitosan-functionalized MWCNTs inhibited BEL-7402 tumors growth by 75% to 52% for pure doxorubicin. Curcumin-loaded nano-cocoons were formulated by vortex mixing a MWCNT-PEG solution with tungsten carbide balls. Experiments indicated that these nano-cocoons were hemo-compatible, non-toxic in mouse fibroblast (L929) cells, and produced an increase in the uptake of curcumin onto rat brain cancer cells (C6 glioma) [113]. Doxorubicin can be successfully delivered to HepG2 cancer cells via galactose-conjugated multiwalled carbon nanotubes, with pH-dependent drug release. In vitro, galactose-conjugated MWCNTs had significant tumor-targeting properties, with a higher cellular uptake efficiency in HepG2 cells, compared to free doxorubicin and had acceptable biocompatibility. Furthermore, in vivo studies in mice implanted with H22 cancer cells indicated that doxorubicin-loaded galactose-conjugated MWCNTs accumulated to a higher level in the tumors, compared to free doxorubicin [114]. The uptake of amphotericin B-loaded mannosylated MWCNTs was increased in J774 macrophages and macrophage-rich organs, compared to pure amphotericin B, highlighting the potential applications for leishmaniasis-targeted drug delivery [115]. Fucose-modified MWCNTs were effective in delivering sulfasalazine to Kupffer cells for the treatment of cytokine-induced liver injury. The sulfasalazine-loaded fucosylated MWCNTS, compared to free sulfasalazine, had enhanced drug loading (>80%), sustained drug release, decreased haemolytic toxicity, increased efficacy in J774 macrophage cells, greater inhibition of IL-12 p40 levels and suppression of nuclear factor–κB [116]. Doxorubicin-loaded dexamethasone-functionalized MWCNTs had a greater efficacy in A-549 lung epithelial cancer cells, compared to free doxorubicin [117]. Glycyrrhizin-conjugated polypropylene imine (PPI) dendrimers and glycyrrhizin-conjugated MWCNTs loaded with doxorubicin produced a 2-fold increase in the delivery of doxorubicin to liver cancer cells [118]. Joshi et al. [119] reported that methotrexate (MTX), when conjugated with aminated C60-fullerenes/MWCNTs, was efficiently delivered to MDA-MB-231 breast cancer cells. The MTX-linked aminated MWCNTs/fullerenes had a tumor pH- sensitive drug release, increased uptake into tumors, and acceptable biocompatibility. The results also suggest that C60-fullerenes have a better drug carrier profile than MWCNTs [119].

Prostate homing peptide-functionalized oxidized MWCNTs encapsulating doxorubicin induced a specific cytotoxicity in metastatic prostate cancer cells (LNCaP). These functionalized MWCNTs had intra-tumoral pH-sensitive drug release and efficacy in LNCaP metastatic prostate cancer cells LNCaP that was comparable to free Dox, indicating the feasibility of these nanoconstructs for anticancer drug delivery [120]. Methotrexate (MTX) tethered to multi-walled carbon nanotubes by various cleavable linkers with amino functionality, was efficacious in human breast cancer cells, and the potency was dependent on the type of linker used for the conjugation of MTX [121]. PEGylated oxidized MWCNTs modified with angiopep-2 have been used to develop a delivery system for targeting brain glioma. The synthesized MWCNTs had an ultrahigh surface area, high loading of doxorubicin, distribution in the brain, and the potential to accumulate in tumors [122].

MWCNTs functionalized with chondroitin sulphate and α-tocopheryl succinate (α-TOS–CSH–MWCNTs) were fabricated for the CD44-receptor delivery of doxorubicin to triple negative breast cancer cells. Doxorubicin-loaded α-TOS-CSH-MWCNTs had increased cellular uptake, stability and hemocompatibility amd greater efficacy, compared to pure doxorubicin [123]. The targeted delivery of doxorubicin to MDA-MB-321 cells, using hyaluronic acid and α-tocopheryl succinate conjugated multi-walled carbon nanotubes, produced a greater efficacy (GI_50_ = 1.184 µg/mL) and induction of apoptosis (52.69%) in MDA-MB-231 cells, compared to pure doxorubicin (GI_50_ = 2.621 µg/mL and apoptosis rate = 15.34%). Overall, the results suggested that the developed formulation had greater efficacy in MDA-MB-231 cells, due to the interaction of hyaluronic acid with the CD44 receptor, which is overexpressed in MDA-MB-231 breast cancer cells [124]. MWCNT-based multifunctional platforms (CNT-PEI(FITC)-mAb), prepared by the grafting of polyethyleneimine (PEI) onto shorter MWCNTs, fluorescein isothiocyanate (FITC), and prostate stem cell antigen (PSCA) monoclonal antibodies (mAb) had an acceptable biocompatibility uptake in cancer cells overexpressing PSCA and contrast, based on the results of in vitro and in vivo ultrasound imaging experiments. Doxorubicin-loaded CNT-PEI(FITC)-mAb selectively accumulated PC-3 tumor-bearing mice and it inhibited the PC-3 tumor growth [125]. Anti-p-glycoprotein (Pgp)-functionalized MWCNTs, with a distearoyl-sn-glycero-3-phosphoethanolamine-polyethylene glycol5000-maleimide linker, had an excellent intratumor diffusion and Pgp-specific cellular uptake, as well as a potent phototoxicity in tumor spheroid-composed MDR cancer cells [126]. 

In summary, research on the use of MWCNTs in drug delivery indicate that functionalized MWCNTs are safe and effective nanocarriers for the targeted delivery of anticancer drugs for cancer therapy.

### 2.7. MWCNTs for Biosensor Applications

MWCNT-based biosensors have been designed for a variety of cancer diagnostic applications. MWCNTs are excellent platforms for the development of MWCNT-based biosensors due to their surface functionalization capacity. Therefore, MWCNTs can be surface-functionalized with antibodies, aptamers, and other molecules for the fabrication of biosensors that can be used for monitoring treatment progress, development of resistance, and detection of antigen levels in cancer patients. Histidine-functionalized MWCNT biosensors have been utilized to detect prostate specific antigens in patients with prostate cancer [127]. A biosensor for detecting prostate cancer was synthesized using multi-wall carbon nanotubes (MWCNTs) conjugated with an antibody against a prostate specific antigen (PSA) using bovine serum albumin. This biosensor was successful in detecting prostate cancer at an early stage, thereby making it valuable for cancer detection [128]. Amino-functionalized MWCNTs conjugated with a monoclonal anti-PSMA antibody was used to develop an electrochemiluminescence ELISA-like immunosensor for detecting PSMA-positive prostate cancer cells [129]. Ovarian cancer cells that express the carcinoma-125 antigen can be detected with a label-free immunosensor, based on biofunctionalized MWCNT embedded ZnO nanofibers This immunosensor had a high sensitivity for detecting carcinoma-125 antibodies in ovarian cancer cells [130]. In whole blood, the cancer antigen, CA 19-9, was detected using a lateral flow strip biosensor (LFSB) and a magnetized carbon nanotube (MCNT) immobilized with the anti-carbohydrate antigen 19-9 (CA 19-9) antibody [131]. The drug, abiraterone, which is used to treat metastatic castration resistant prostate cancer [132], was detected using a CYP3A4 protein-conjugated MWCNT enzymatic biosensor. The developed biosensor detected abiraterone at very low concentrations in cancer patients [132]. The consumption of L-tryptophan by cancer cells in the extracellular matrix can be used as a prognostic marker for metastasis and can be detected using a L-tryptophan aptamer noncovalently attached to MWCNTs [133]. To detect human leukemic lymphoblasts (CCRF-CEM), researchers developed a flow injection amperometric sandwich-type aptasensor made from gold nanoparticle-decorated poly(3,4-ethylenedioxythiophene) to immobilize thiolated sgc8c aptamer and palladium nanoparticles/3,4,9,10-perylene tetracarboxylic acid-decorated MWCNTs [134]. An ultrasensitive FET-CA125 aptasensor was synthesized by attaching the ovarian cancer antigen CA-125 to a field effect transistor (FET), composed of MWCNTs and reduced graphene oxide (rGO) [135]. Sandwich-type electrochemical aptasensors for detecting adenocarcinoma gastric (AGS) cancer cells in human serum were synthesized by immobilizing the primary thiolated aptamer and secondary aptamer-Au@Ag nanoparticles on MWCNT-Aunano nanoplatforms [136]. Nanomaterials, based on MWCNTs are effective for the design of biosensors for the early and late detection of various types of cancers. MWCNT-based biosensors are therefore effective sensors for the detection of various cancers in the early stages [137,138].

## 3. Conclusions

Multi-walled carbon nanotubes, which have excellent mechanical, physical, and unique drug transport properties, represent an important class of nanomaterials that have significant potential in the areas of engineering and nanomedicine. Despite their wide applicability, the utility of MWCNTs is limited by their tendency to self-aggregate and poor dispersibility in different media. The functionalization of MWCNTs is an effective strategy, not only to overcome the problem self-aggregation and poor dispersibility, but also to increase their biocompatibility and aqueous solubility, thereby facilitating their use as nanocarriers in nanomedicines. However, the functionalization of carbon nanotubes frequently involves chemical processes that use toxic chemicals, toxic catalysts, and harsh synthetic conditions. Microwave chemistry, which decreases reaction time and limits the demand on the use of solvents, offers an environment friendly method for the functionalization of MWCNTs, with improved yields, compared to conventional methods. Microwave irradiation has been used to introduce functional groups, such as carboxylic acid group and epoxy groups, from the carbon atoms of the MWCNTs, which then can be used for the conjugation of vitamins, proteins, and polymers. Microwave-assisted functionalization of MWCNTs involves the noncovalent adsorption and covalent attachment of biomolecules, such as lysozymes, dopamine, arginine, amino uracil, and riboflavin. This type of functionalization establishes MWCNTs as versatile nanomaterial with practical applicability in areas such as drug delivery, biosensor development, and organocatalysis, among others. Despite the challenges associated with the scaling up of chemical processes using microwave technology, the microwave-assisted functionalization of MWCNTs is a promising, environmentally friendly methodology, in terms of high productivity, and thus will advance the application of MWCNTs in engineering, nanomedicine, and drug delivery, as is evident from the literature discussed in this review.

## Data Availability

Not applicable.
